# Supplementary material for: Analysis of cholesterol in mouse brain by HPLC with UV detection
Source: PLoS One. 2020 Jan 24;15(1):e0228170. doi: 10.1371/journal.pone.0228170 (PMC6980532; doi:10.1371/journal.pone.0228170)
Supplement: Attachment — Submitted filename: Comments-pLOS ONE.docx [file pone.0228170.s001.docx]

In this manuscript, the authors María Alejandra Paulazo & Alejandro Omar Sodero proposed a RP-HPLC-UV method for the analysis of cholesterol in mouse brain. The experimental design and the result are reasonable and reliable.

However, there are some points which need to be further considered.

Major comments:

1. At the end of the introduction (P4), authors need to give the other literature reports of the cholesterol determination by instrumental techniques.
2. As the authors developed and validated a RP-HPLC method for cholesterol, it is suggested to perform and include the other validation parameters like system suitability, specificity, ruggedness, robustness, etc.
3. Provide the data table(s) which includes the chromatographic and validation parameters.
4. Whether the authors cross-checked the peak purity of the cholesterol peak at RT 8.4 in Figure 2? Because as the sample run contains many peaks due to impurities in it, there are all the possibilities that the other impurities peaks may overlapped on it. It can be overruled by examining the peak purity of cholesterol (with the help of chromatographic software).

Minor comments:

1. P2, line 20; Abstract: HPLC to be expanded instead of in line 22.

5. P4, line 64-65, authors are claiming that the proposed method is in agreement with previous reports; they need to provide the literature for the previous reports.
6. P4, line 75, make +2 state of Ca/Mg superscript.
7. P5, line 96, authors claiming temperature was set at 28^o^C. It is not clear about the temperature of which was set at 28^o^C. Be clear about it. I think it is the column temperature.
8. P7, line 128, make 2 of R2 superscript.
9. P8, line 158, the sentence after the reference [18], is not correct.
10. P11, line 220, make 2 of R2 superscript.
11. Mention the name and RT on the cholesterol peak in the Figures 1 & 2.
