# Supplementary material for: Analysis of cholesterol in mouse brain by HPLC with UV detection
Source: PLoS One. 2020 Jan 24;15(1):e0228170. doi: 10.1371/journal.pone.0228170 (PMC6980532; doi:10.1371/journal.pone.0228170)
Supplement: Attachment — Submitted filename: Response to reviewers.docx [file pone.0228170.s002.docx]

Dear Editor and reviewers,

I very much appreciate the criticisms and suggestions, which have led to this revised version of our manuscript entitled “Analysis of cholesterol in mouse brain by HPLC with UV detection”.

I bellow answer point-by-point the major and minor comments. Please, note all the responses in red.

Reviewer # 1:

In this manuscript, the authors María Alejandra Paulazo & Alejandro Omar Sodero proposed a RP-HPLC-UV method for the analysis of cholesterol in mouse brain. The experimental design and the results are reasonable and reliable. However, there are some points which need to be further considered.

Major comments:

1. At the end of the introduction (P4), authors need to give the other literature reports of the cholesterol determination by instrumental techniques.

We have added in Page 4 the references describing other instrumental techniques.

2. As the authors developed and validated a RP-HPLC method for cholesterol, it is suggested to perform and include the other validation parameters like system suitability, specificity, ruggedness, robustness, etc.

We have included in the Materials & Methods section the omitted validation parameters: matrix effect, intra- and inter-day precision (Table 1) and robustness.

3. Provide the data table(s) which includes the chromatographic and validation parameters.

We have added Table 1, showing the intra- and inter-day RSD at five different cholesterol concentrations.

4. Have the authors cross-checked the peak purity of the cholesterol peak at RT 8.4 in Figure 2? Because the sample run contains many peaks due to impurities in it, there are all the possibilities that the other impurities peaks may overlapped on it. It can be overruled by examining the peak purity of cholesterol (with the help of chromatographic software).

Concerning the possibility of impurities in the cholesterol peak with RT = 8.4, we can say that our Chromeleon software quantifies the signal as a single peak, although in same matrices (brain but not cells) and at certain dilutions, we can appreciate a small unidentified shoulder just before the absorbance starts to increase. Our interpretation is that in brain samples the area of the cholesterol peak at RT = 8.4 is big enough in comparison to the descending half of the unidentified shoulder area, therefore the small shoulder area does not significantly influence the cholesterol quantification.

Minor comments:

1. P2, line 20; Abstract: HPLC to be expanded instead of in line 22.

Now it reads high performance liquid chromatography (HPLC).

2. P4, line 64-65, authors are claiming that the proposed method is in agreement with previous reports; they need to provide the literature for the previous reports.

We have included the reports in Page 4 and in the reference list.

3. P4, line 75, make +2 state of Ca/Mg superscript.

It was corrected.

4. P5, line 96, authors claiming temperature was set at 28^o^C. It is not clear about the temperature of which was set at 28^o^C. Be clear about it. I think it is the column temperature.

It was corrected. Indeed, we were refereeing to the “column temperature”.

5. P7, line 128, make 2 of R2 superscript.

It was corrected.

6. P8, line 158, the sentence after the reference [18], is not correct.

We agree with this comment. We have now softened the sentence and reads “… represents a reliable alternative to measure cholesterol in biological samples”.

7. P11, line 220, make 2 of R2 superscript.

It was corrected.

8. Mention the name and RT on the cholesterol peak in the Figures 1 & 2.

We now say in the figure legends that the cholesterol peak is the colored one.

Reviewer # 2:

HPLC analysis of cholesterol in biological samples had long been reported and the manuscript described a traditional method with limited analytical performance. Additionally, the validation of the method was insufficient. The presented data did not support the claim of "very good precision and accuracy". A recovery of 74% might be too low for cholesterol analysis. The specificity of the method was unknown.

We do not fully agree with this reviewer. We believed that our method shows good to very good analytical performance (instead of limited). Now, we present additional data that sustain the claim of “very good precision and accuracy”. The intra- and inter-day RSD were below 5%, and the analyte recovery increased to 85.7 ± 10.9 %, after increasing the number of assays from 1 to 3. This recovery level is widely accepted in the lipids field.

NOTE: As required by the Editor, we have included a description of the method utilized for mouse sacrifice (Page 4, Materials & Methods), and Table 2 (Page 18) that highlights the main differences with other analytical approaches already published. In addition, we have included a new version of the Figure 3 made with the Prism software, and formatted the manuscript following the journal’s guide.

Clarification of the Editor to a particular authors’ inquiry:

The main problem with UV detector is the possibility to have interfering compounds eluting at the same retention time as the target analyte. For this reason, the sample treatment and the chromatographic separation should be carefully developed to avoid such problem. Using DAD detector and particular software tools, it is possible to evaluate the chromatographic peak purity (see the following link, https://www.agilent.com/cs/library/applications/5988-8647EN.pdf). In case of a single wavelength UV detector (as your system), the presence of a shoulder, valley or excessive tailing of your peak may suggest the suspected of the presence of an impurity. Thus, considering these aspects, with LC-UV based-methods is mandatory to a) evaluate the matrix effect by comparing the slope of calibration curves prepared in both standard and matrix samples and b) compare the LC-UV method with LC-MS, analyzing real samples spiked with a known amount of the target analyte. So, I suggest to carry out these latter experiments to validate your LC-UV method. For this reason, I suggest to have a look of these articles:

DOI: 10.1371/journal.pone.0028182

DOI: 10.1016/j.chroma.2013.08.091

DOI: 10.1371/journal.pone.0114430

We acknowledge the Editor for the suggested literature. It was really helpful to improve our manuscript. As suggested by the Editor, we have checked for a possible matrix effect and couldn’t find any significant difference between the slopes of the curves made by spiking different amounts of cholesterol in mobile phase or brain matrix (see below Figure for the Editor). Unfortunately, at the moment, we don’t have a mass detector coupled to our HPLC system to do the required comparison between LC-UV and LC-MS. That’s the reason why we setup this UV-based method to assess cholesterol in biological samples.

Figure for the Editor. Average slope ± SD of cholesterol standard curves made either in mobile phase (n = 7) or brain matrix (n = 4). The statistical analysis revealed no significant differences between the average slopes of both groups (7.82 ± 1.06 vs. 8.06 ± 1.59; p = 0.80, Student’s t-test), meaning there is no matrix effect.

Hoping to hear from you,

Best regards,

Dr. Alejandro O. Sodero
